# Current recommendations/practices for anonymising data from clinical trials in order to make it available for sharing: A scoping review

**DOI:** 10.1177/17407745221087469

**Published:** 2022-06-22

**Authors:** Aryelly Rodriguez, Christopher Tuck, Marshall F Dozier, Stephanie C Lewis, Sandra Eldridge, Tracy Jackson, Alastair Murray, Christopher J Weir

**Affiliations:** 1 Edinburgh Clinical Trials Unit, Usher Institute of Population Health Sciences and Informatics, The University of Edinburgh, Edinburgh, UK; 2 Centre for Cardiovascular Science, The University of Edinburgh, Edinburgh, UK; 3 Library & University Collections, Information Services, The University of Edinburgh, Edinburgh, UK; 4 Pragmatic Clinical Trials Unit, Blizard Institute, Barts and The London School of Medicine and Dentistry, Queen Mary University of London, London, UK; 5 Asthma UK Centre for Applied Research, Usher Institute of Population Health Sciences and Informatics, The University of Edinburgh, Edinburgh, UK; 6 Independent Researcher, Edinburgh, UK

**Keywords:** Clinical trials, systematic review, data anonymisation, patient identification systems, personally identifiable information, datasets, data curation, guidelines

## Abstract

**Background/Aims:**

There are increasing pressures for anonymised datasets from clinical trials to be shared across the scientific community, and differing recommendations exist on how to perform anonymisation prior to sharing. We aimed to systematically identify, describe and synthesise existing recommendations for anonymising clinical trial datasets to prepare for data sharing.

**Methods:**

We systematically searched MEDLINE^®^, EMBASE and Web of Science from inception to 8 February 2021. We also searched other resources to ensure the comprehensiveness of our search. Any publication reporting recommendations on anonymisation to enable data sharing from clinical trials was included. Two reviewers independently screened titles, abstracts and full text for eligibility. One reviewer extracted data from included papers using thematic synthesis, which then was sense-checked by a second reviewer. Results were summarised by narrative analysis.

**Results:**

Fifty-nine articles (from 43 studies) were eligible for inclusion. Three distinct themes are emerging: anonymisation, de-identification and pseudonymisation. The most commonly used anonymisation techniques are: removal of direct patient identifiers; and careful evaluation and modification of indirect identifiers to minimise the risk of identification. Anonymised datasets joined with controlled access was the preferred method for data sharing.

**Conclusions:**

There is no single standardised set of recommendations on how to anonymise clinical trial datasets for sharing. However, this systematic review shows a developing consensus on techniques used to achieve anonymisation. Researchers in clinical trials still consider that anonymisation techniques by themselves are insufficient to protect patient privacy, and they need to be paired with controlled access.

## Introduction

Clinical trials are complex, time-consuming and costly, and it is wasteful not to use data fully.^1^ Therefore, when academic-led clinical trials are completed, their results are usually released to the public and wider scientific community in scientific journals or clinical trials registries. Existing clinical trials’ data can be used to answer novel clinical questions, to reproduce and check analysis, to understand basic science, to investigate new methodologies and for teaching.^2^ Also, there are sometimes considerable amounts of data that are not analysed as part of the published results.^3^ In addition, trial data are often useful after the end of a trial to perform meta-analyses across several trials and using the individual patient data from each trial adds to the quality of such analyses,^4^ for instance, by allowing full investigation of subgroup effects. There is now a drive, particularly from publishers and funders, to encourage the general release of relevant anonymised trial datasets^5^ among interested parties.

Clinical trial datasets contain personal health information of the trial participants. It is imperative that data sharing does not disclose personal data to anyone who falls outside the original group to whom the trial participants have provided consent to access their data. Anonymising the trial dataset fulfils this requirement. However, the anonymisation process removes information from the data, and if not done carefully, the original trial analyses could not be reproduced, which in turn will limit the data’s usability for further research.^6^

The drive to share data more widely has generated various sets of recommendations to enable sharing.^5,7–10^ Embedded within these, there is a variety of recommendations on how to anonymise a dataset.

### Why it is important to do this review

To our knowledge, there are no reviews of the methods and/or recommendations for the process of generating anonymised clinical trial datasets (a search was executed on the 15 February 2021 on Google Scholar^11^ with ‘literature’ ‘review’ ‘anonymization’ ‘methods’ ‘clinical trials’ and also ‘literature’ ‘review’ ‘anonymisation’ ‘methods’ ‘clinical trials’, the first 100 results were screened for each search and relevant results were not found).

To understand and collate the techniques used or recommended for data anonymisation in clinical trials, a systematic scoping review is required.

### Objective

To identify, describe and synthesise the existing methods/recommendations to anonymise datasets from clinical trials.

## Methods

The *Joanna Briggs Institute Reviewers’ Manual: 2015 Methodology for JBI Scoping Reviews* ^12,13^ and the PRISMA Extension for Scoping Reviews (PRISMA-ScR)^14^ were followed for the execution of this scoping review.

### Types of publications

We included any publications or documentation giving recommendations on anonymising datasets from clinical trials in any therapeutic area. Non-empirical publications, such as editorials, expert views or practice guidelines were also included in this review

### Type of outcomes

The primary outcome is the reported methods and/or recommendations for anonymisation of clinical trials datasets.

### Search methods for identification of publications

We performed a comprehensive systematic search to identify publications reporting methods or recommendations for anonymising clinical trials datasets. No language restrictions were imposed to attempt worldwide coverage. We did not identify any non-English publications.

#### Electronic searches

Web of Science (WoS), MEDLINE^®^ (including non-indexed and in-process records) and EMBASE databases were searched from inception to 11 February 2019. The searches were rerun from 1 January 2019 to 8 February 2021 for MEDLINE^®^ and EMBASE. A discrepancy was identified by M.F.D. in the original WoS strategy, so that, we reran the complete search from inception to 8 February 2021.

The search strategy used the following key concept areas, adopting subject headings and keywords as relevant for each database:

(Clinical) and

(trial* or randomi* or research* or control*) and

(principle* or guid* or recomm*) and

(shar* or reus* or re-us* or access* or open) and

(de-identi* or deidenti* or anonym* or privacy or confidential*)

The search was piloted with four indicator papers (Ohmann,^5^ Keerie,^9^ Tudor-Smith^15^ and Hrynaszkie-wicz^16^) that the searches needed to retrieved to ensure their effectiveness. The resulting detailed electronic search strategies are presented in Appendix 2 in the supplemental materials.

#### Searching other resources

To ensure the comprehensiveness of our search, we searched the websites of major research governance organisations and public research funding bodies as recommended by the Health Research Authority^17^ and the Wellcome Trust,^18^ the top 10 wealthiest charities,^19^ the top 10 UK charities by brand value^20^ and all registered UK academic clinical trials units,^21^ to find guidelines published as grey literature from February 2019 until March 2020, so as not to omit documents not published as journal articles and not indexed in the bibliographic databases.

To further supplement our search field, we used citation and reference tracking (backwards and forward citation searching) on the selected articles from the electronic searches in order to identify additional sources. Preliminary results of this project were presented at the Fifth International Clinical Trials Methodology Conference 2019^22^ where we requested to be contacted by any author or expert who could assist with the project but we did not receive any replies. During this event, several colleagues suggested publications to include in our grey literature.^23,24^ Shortly after, the COVID-19 pandemic started and we decided not to burden authors/experts with our requests and to concentrate on getting this project executed with the evidence that we had already collected. All the items included in this review obtained via the search of other resources were re-checked on May 2021 to locate updated versions since the original search.

### Data collection and analysis

Records were retrieved and transferred into the reference manager EndNote,^25^ which was used for de-duplication and to maintain a master library of the records throughout the review process. Covidence software^26^ was used for further de-duplication, screening and full-text review. Two reviewers (A.R. and either C.T. or A.M.) independently screened titles and abstracts for eligibility. Full-text copies of all potentially relevant records were obtained using the reference manager.

Records identified from citation and reference tracking, and major research governance organisations, public research funding bodies and charity websites were collated in MS Excel,^27^ for manual de-duplication and title screening. Records selected for full-text review were manually retrieved. Two teams (A.R. and either C.T. or A.M.) independently assessed whether each full-text record met the inclusion criteria. Chosen full-text records were added to the master library in EndNote.^25^

Any discrepancies were discussed between the reviewers and if agreement could not be reached then it was arbitrated by a third reviewer (S.C.L., C.J.W. or S.E.).

Publications were excluded if they did not have concrete recommendations/methods of anonymisation, or they were not from a clinical trial framework, or they were focused on omics data or big data.

#### Data extraction/management and synthesis

A data extraction form to collect relevant data items from eligible sources was developed and piloted in line with Cochrane guidance,^28^ this included: publication details (Authors names, Journal, year), country and classification (from electronic search or from other sources).

Data extraction and analysis was undertaken by one reviewer (A.R.) in NVivo^®29^ using thematic synthesis.^30,31^ Therefore, the included records were read‘line-by-line’, and when recommendations/methods on anonymisation were found, they were coded to a theme. At this stage, we allowed themes to be free and data-driven (i.e. to emerge from the data), rather than rigidly defining them a priori. It was possible to assign several themes to the same sentence. An independent sense-check was conducted by a second reviewer (A.M.) of the free themes. Any discrepancies were discussed between the reviewers and if an agreement could not be reached then it was resolved by a third reviewer (S.C.L., C.J.W. or S.E.).

The free themes were grouped into broader themes by the study team, this was repeated until we reached a final theme structure. We did not attempt to generate analytical themes^30^ as our goal was to only identify the existing recommendations/methods on anonymisation.

Finally, the data from the included publications were summarised in descriptive tables. Themes were summarised by narrative analysis^32^ and if applicable descriptive statistics.

## Results

**Figure 1.**
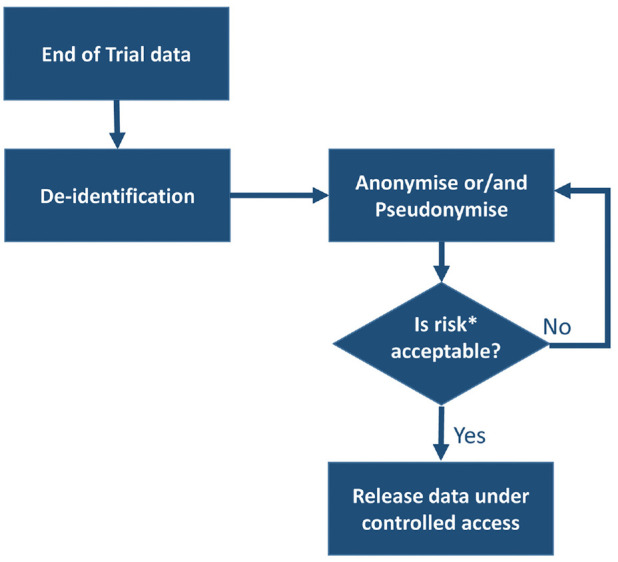
Most suggested method to release anonymised datasets from clinical trials. Risk of re-identification is a complex variable, which is minimised using controlled access. The description of risk is out of scope for this review. Other processes: five studies described usage of open access instead, one study mentioned both controlled and open access for data release and the remaining two studies did not discussed data release.

We identified 1059 potentially eligible records (Figure 1 in the online supplemental materials). Six hundred thirty-seven records were excluded after title and abstract screening. Three hundred sixty-three records were excluded after full-text review. Fifty-nine records^5,9,15,16,23,24,33–86^ (representing 43 studies) met the inclusion criteria and were included in the final qualitative synthesis (Appendix 3 has the full list and characteristics of the included records).

### Included studies’ characteristics

Table 1 summarises the observed characteristics of the included studies and their associated records, it also shows the included studies by source and country/region and year of publication. Figure 2 in the online supplemental materials shows the included studies over time.

**Table 1.** Studies/record characteristics.^a^

Parameter	Category	Studies *N* = 43, *n* (%)	Records *N* = 59 *n*(%)
Source^b^	Electronic search	19 (44)	21 (36)^c^
	Other sources	24 (56)	38 (64)^d^
Country/region	EU	12 (28)	24 (39)
	UK	11 (26)	14 (23)
	US	10 (23)	12 (20)
	Canada	5 (12)	5 (8)
	Australia	2 (5)	2 (3)
	US–EU–UK	2 (5)	3 (5)
	South Korea	1 (2)	1 (2)
Year of publication	2003–2008^b^	5 (12)	5 (8)
	2009–2014^b^	15 (35)	17 (29)
	2015–2020^b^	23 (53)	37 (63)
Studies split by source				
Parameter	Category	Electronic search *N*=19, *n* (%)	Other sources *N*=24 *n*(%)
Studies split by country/region	Canada/US	6 (32)	9 (37)
	EU/UK	12 (63)	11 (46)
	Other regions^e^	1 (5)	4 (17)
Studies split by year of publication	2003–2008^f^	3 (16)	2 (8)
	2009–2014^f^	7 (37)	8 (33)
	2015–2020^f^	9 (47)	14 (58)

a Therapeutic field was not applicable and it was not recorded.

b Where applicable, the oldest record in the included study determined the overall study date.

c Corresponding references^5,9,15,16,33–46,48,49,87^

d Corresponding references^23,24,50–84,86^

e Consisting of Australia, the United States–EU–the United Kingdom and South Korea.

f Where applicable, the oldest record in the included study determined the overall study date.

### Deriving the coding themes

A NVivo^®^ exploratory word cloud was generated, it displayed the frequency in which significant words appeared in the included studies from the electronic searches (Figure 3 in the online supplemental materials), and it provided an initial idea of the themes present in the available data.

A.R. started the coding into free themes. As the actual coding progressed, the themes were reviewed and grouped by the study team until its structure was locked on 5 September 2019 by A.R., S.C.L. and C.J.W. The subsequent coding of the studies from other sources did not add any new themes. Eleven themes were identified (see Table 1 in the online supplemental materials).

### The body of knowledge after coding themes

The 11 themes were applied to all 43 included studies (see Table 2). The most common theme among the selected studies were the definitions of de-identification (34 studies (79%)), anonymisation (28 studies (65%)), techniques for the manipulations of data (34 studies (79%)) and the implementation of controlled access for data release (38 studies (88%)).

**Table 2.** Themes by studies.

Id/theme	Studies (*N* = 43)		Associated records
	*n*	%	
1. Anonymisation	28	65	5, 15, 16, 33, 34, 36, 37, 42, 44–48, 50–54, 56, 57, 63, 65, 66, 68–71,77–80, 82, 83, 85–87
2. De-identification	34	79	5, 15, 16, 33, 34, 36, 37, 42, 44–48, 50–54, 56, 57, 63, 65, 66, 68–71,77–80, 82, 83, 85–87
2.1. HIPAA identifiers^a^	23	53	5, 16, 24, 33, 34, 36–39, 43, 46, 47, 52–54, 56, 57, 59, 60, 63, 65, 66, 76,77, 80, 82–87
2.2. Hrynaszkiewicz identifiers^b^	12	28	9, 16, 33, 45–47, 59, 60, 63, 66, 74, 78, 85–87
3. Pseudonymisation	23	53	5, 9, 34, 36, 37, 40–44, 46, 49–51, 55–57, 66, 68, 69, 71, 77, 82
4. Manipulation of data	34	79	9, 15, 16, 24, 33, 36–38, 42–48, 50, 53, 54, 56, 57, 59, 61, 62, 64–66,69, 71, 75, 77, 78, 80–84, 87
4.1. Perturbation^c^	7	16	9, 36, 55, 66, 67, 77, 84
4.2. Recalculation^c^	12	28	9, 16, 23, 43, 45, 52, 54, 56, 59, 63, 64, 67, 70, 73, 78, 80, 82, 83
4.3. Recoding^c^	16	37	9, 33, 35, 43, 51–55, 59, 60, 63, 64, 66, 67, 69, 70, 72, 77–79, 82–84
4.4. Suppression^c^	17	39	9, 35, 45, 51–54, 56, 57, 59, 60, 62, 63, 65–67, 69, 70, 72, 73, 77,78, 80, 82–84
4.5. Remove superfluous data^c^	2	5	45, 48
5. Privacy model	12	28	35, 38, 40–42, 46, 55, 57, 66, 69, 84–86
5.1. K-anonymity^c^	7	16	35, 38, 40, 55, 57, 69, 84
6. Controlled access	38	88	5, 9, 15, 16, 24, 33, 34, 36–39, 44–48, 50, 54, 57, 59, 60, 62–66, 71,72, 75, 77–87
6.1. Black box^c^	3	7	41, 43, 46
6.2. Encryption^c^	8	19	36, 39–42, 57, 66, 77
6.3. Safe haven^c^	8	19	33, 36, 43, 46, 47, 55, 66, 83, 87
6.4. Split location^c^	5	12	34, 41, 43, 66, 81
7. Open access	7	16	9, 15, 36, 50, 56, 66, 85, 86
8. Central repositories	5	12	16, 33, 46, 62, 66
9. Expert determination	12	28	16, 24, 38, 46, 51, 66, 65, 74, 76, 77, 80, 82–84
10. Provision of context documents	12	28	5, 9, 15, 44, 46–48, 62–64, 66, 75, 79, 82, 83, 87
11. Risk calculation	15	35	16, 33, 35–37, 47–49, 57–59, 66, 69, 72, 78, 81, 82, 84–87

HIPAA: Health Insurance Portability and Accountability Act.

a HIPAA identifiers refers to the HIPAA Safe Harbor method that requires the removal of 18 items of protected health information.^76^

b Hrynaszkiewicz identifiers refers to the removal of direct identifiers (information sources such as name and/or address, which on their own canre-identify participants) and the consideration/removal of indirect identifiers (variables that on their own might not represent a risk of re-identification for participants but in combination with of other indirect identifiers might increase the risk of re-identification, e.g. sex combined with age).^16^

c These are child codes that are included in their parent code.

In general terms, when study authors described anonymisation, de-identification and pseudonymisation, their explanations gravitated around the definitions presented in Table 3.

**Table 3.** Most common definitions for anonymisation, de-identification and pseudonymisation.

Pseudonymisation	De-identification	Anonymisation	
• Attributes are replaced with pseudonyms on a one-to-one correspondence • It is never an effective means of anonymisation • A security enhancing measure • Pseudonyms bear no relationto the patient details • Preferably reversible	• Stripping datasets of patients identifying variables as per either: ○ HIPAA 18 items‘Safe Harbor’ method (US) ○ Hrynaszkiewicz et al.28 items of personal andclinical information (Europe)	• Any given record lacks any individuality,distinction or recognisability • Can potentially distort data • The link with the original dataset should be destroyed • Set at a level to reach acceptable risk, but binary in law	
Most common definitions for data manipulation techniques^a^				
Suppression (removal, elimination)	Recoding (grouping, masking, replacement, generalisation, blurring, aggregation)	Recalculation	Perturbation
• Delete outliers • Delete free-text • Delete high-risk variables • Delete high-risk records	• Keep first three digits of postcode • Categorise age (18–40) and >= 40	• Show age instead of DOB • Show study day relativeto randomisationday, instead of date(e.g. day 7) • When dates are important they are presented offset	• Add randomnoise to variables • Replace data withsimulated randomvalues • Data shuffling • Rounding of variables

HIPAA: Health Insurance Portability and Accountability Act.

^a^Tuck et al.^45^ and Tudur-Smith et al.^48^ mentioned the removal of superfluous data (e.g. deletion of data, such as audit trails) to supplement data manipulation techniques.

The described aim of data manipulation is to transform variables to reduce detail, without taking away too much data utility. The most common data manipulation methods’ definitions are given in Table 3.

Twelve studies (28%) recommended the use of privacy models (such as k-anonymity,^88^ l-diversity^89^ and differential privacy^90^) to further guarantee and assess data anonymity to protect datasets from re-identification attacks.

The theme of controlled access mostly referred to the implementation of data-sharing agreements, the location of data behind a secure access barrier (either physical, virtual or both), the identification and vetoing of secondary research (e.g. checking requesters are bona fide researchers with a valid research question). In contrast, the theme of open access referred to minimal (or non-existent) requirements for allowing access to the data set to secondary researchers.

Central repositories (mentioned by five studies (12%)) were described as destinations where institutions could deposit their datasets to be managed by a third party and accessed by secondary researchers.^92–95^

The expert determination method for dataset release (12 studies (28%)) was generally described as when an expert (chosen for their knowledge/qualification) could assess the risk of re-identification of clinical trial datasets using ‘generally accepted statistical and scientific principles’,^66^ if the risk is low, the data are certified and granted release to a secondary researcher.

Twelve studies (28%) recommended the provision of documental context to avoid erroneous interpretation and use of the anonymised datasets. Suggested documents to be provided included: original study protocol (and applicable amendments), statistical analysis plan, annotated case report forms and a data dictionary.

Finally, 15 studies (35%) highlighted the importance of assessing the risk of the anonymised dataset before making a decision on release, however, only four records^35,59,66,69^ (three studies) described how the risk could be calculated.

### Most suggested processes for sharing anonymised datasets

Thirty-five studies (81%) described that at the end of a clinical trial, data should be de-identified (key items stripped from the dataset). Following this, data manipulation techniques should be used to further anonymise the datasets. Finally, the datasets should be made available under a controlled access approach.

Thirteen of those 35 studies also mentioned a step before release under controlled access in which the risk of re-identification should be assessed. This would start an iterative process, and once the risk is deemed acceptable, the anonymised data set should be made available under controlled access (Figure 1).

## Discussion

The EU/UK region provided 53% of the included studies, followed by the US/Canada region with 35%, while the rest (12%) originated from other regions. This result was very similar when studies were split by source. Similarly, 53% of the included studies were published after 2015, 35% of the studies were published from 2009 to 2014 and the rest (12%) of the studies were published from 2003 to 2008. This profile was also observed when the studies were split by source, this shows the greater interest in this topic as time progresses. Overall, the EU/UK region from 2015 to 2020 was the most prolific with 16 studies out of 43 (37%). Where the content in the included studies was congruent regarding the source of the studies, this was noted, while the studies from other sources were coded because there was no need to update the coding themes generated with the studies from the electronic searches. However, a small but crucial difference is that studies from other sources have more detail and examples regarding data manipulations; this is most probably due to the lack of restriction on publication size for this type of source.

*Topic 1: The relationship among the themes, pseudonymisation, de-identification and anonymisation, in the context of clinical trials*. Anonymisation versus de-identification: they are both described as tools to facilitate data sharing. They rarely appear in isolation in any of the included studies, because they are part of the wider theme of data transparency and patient privacy. In this review, seven records coded to anonymisation, 14 records to de-identification and 28 records coded to both themes.

Anonymisation is presented as an abstract theme with lots of interpretation, mostly shaped by the regional laws where the publications originated (i.e. each researcher would have a theme that they favour which is shaped by their legal framework). These laws could be vague with their definitions and this could explain the existence of multiple concepts. On the other hand, de-identification is a more clear-cut and widely harmonised theme because it is defined in a precise way via Health Insurance Portability and Accountability Act (HIPAA).^96^

The themes of anonymisation and de-identification appear to be gradually evolving, for example, older records considered anonymisation and de-identification as equivalent, while newer studies consider de-identification as a mechanical process to remove the identifiers, whereas anonymisation is the next step to prepare data for sharing (via data manipulation and privacy models). In general, most authors adhere to the narrative of further anonymising (via data manipulation and privacy models) the dataset after key variables have been removed, regardless of their previous definition of anonymisation and de-identification. Anonymisation is as a process to balance the minimisation of the probability of re-identification versus the utility of a clinical trial dataset, (e.g. too much anonymisation could render the data unusable).^36,44,46,50,66,69,71,86,87^ Therefore, data cannot be fully anonymised in the context of clinical trials.

Also, it seems well accepted and understood among authors that some variables in a clinical trial dataset are identifiers and that they can be classified as direct (e.g. name or address)^16^ and indirect (also named as quasi-identifiers (e.g. present age instead of date of birth)).^16^

Pseudonymisation of data usually occurs in the initial stages of data collection within clinical trials.^5^ It also has a regional connotation, bound by the local laws and regulations. Pseudonymisation is declared to carry low risk for re-identification,^5,66^ however, no authors from the included studies advocated its use in isolation for data sharing. Some authors acknowledge that pseudonymisation alone is not acceptable for data sharing, as the one-to-one correspondence with the original fully identified dataset still exists, which makes it personal information under the EU and UK General Data Protection Regulation (GDPR).^34,36,42,57,66^

*Topic 2: Most common data manipulation techniques to achieve anonymisation*. Data manipulation techniques can be applied according to the data holder’s preference and technical capabilities and the intrinsic needs of the clinical trial dataset that is being processed.

Data manipulation techniques have multiple names, but there seems to be a progression towards a concerted set of four tools: perturbation, recalculation, recoding and suppression as presented in Table 3, with suppression, recoding and recalculation being the most talked about techniques. Authors are mostly describing via examples what is available regarding data manipulation techniques without critical judgement of the techniques, however, the majority of authors agree that data manipulation techniques are capable of reducing utility if left unexamined.

*Topic 3: The introduction of privacy models*. Clinical trials datasets are relatively small when compared to routinely collected data (e.g. medical records) and the implementation of a privacy model (such as differential privacy^90^) could present challenges, also privacy models could be complicated techniques. This can explain why the uptake of privacy models is modest, despite the fact they come from methodologies that have been tried and tested in big datasets^35,97^ and they could be applied to clinical trials.^35,38,40,55,57^ The most common privacy model mentioned is k-anonymity.^88^

*Topic 4: The importance of controlled access and the tension with open access*. The majority of clinical trial researchers strongly advocate for controlled access to the anonymised datasets, stemming from a concern with correct and genuine use of the anonymised data set.^87,92^

Authors recommend that the secondary researchers should have reasonable research questions and a data-sharing agreement should be put in place, which should include the use of the data for the intended purpose, the implementation of data protection procedures, the prohibition of any patient re-identification, the prohibition of sharing the data with a third party and the acknowledgement of the original authors in the secondary research output.

Regarding the actual sharing of the data, the trend is towards data access (e.g. via a safe haven) instead of data transfer, this means that secondary researchers can see and analyse the dataset but not download it. Here, the central repository plays a key role, because it would prove difficult (when it is necessary), to merge datasets that reside in separate repositories.

It is important to point out that controlled access is not required by laws or regulation, it is something that clinical trials researchers are doing, because it provides better research governance and researchers’ acknowledgement that anonymised datasets are still sensitive.^87^ Stripping identifiers from datasets and the use of manipulation techniques are not sufficient on its own to fully anonymise clinical trials datasets and to protect patient privacy. Understandably, researchers do not want to breach patient trust and they want to pre-emptively defend against a potential data breach and its catastrophic consequences (loss of patient trust, hefty legal fines and loss of reputation),^98^ but they are generally willing to share.^99^

At the other end of the spectrum, open access is a relatively hassle-free release option once the dataset is anonymised, therefore, its existence and practicality is acknowledged, but it is not directly endorsed by any of the included papers as the research governance is very difficult under it. The International Stroke Trial (IST) database^91,100^ which is often cited as an example of a successful open access dataset by authors,^66,87^ also drew criticisms from others^86^ regarding some of the indirect identifiers left in the dataset. However, IST is yet to report a successful re-identification attack. The limited use of open access causes frustration among secondary researchers who are eager to get fast and easy access to datasets.^101,102^

Currently, controlled access is still one of the main cornerstones for the release of anonymised clinical trials data and many authors agree that data should only be released if a threshold of acceptable risk is achieved. There are several available methods for calculating risk, but authors of included studies did not explain sufficiently what ‘acceptable’ means, reasonably, this is very difficult to define as it would depend on the context surrounding the release of the anonymised clinical trials datasets and on the datasets own characteristics.

### Comparison with existing literature

We identified a similar systematic review by Chevrier,^103^ which included all biomedical literature in MEDLINE^®^ between 2007 and 2017. We agreed with them about the existence of multiple interpretations for anonymisation and de-identification and they also discussed the balancing act between the re-identification risk and data manipulation. However, their focus was on electronic health records, and those datasets have different needs and their own challenges when compared with clinical trials datasets.

### Strengths and limitations

Strengths to this review are that the electronic databases were searched since inception without any language restrictions and there was a thorough coverage of grey literature. The database searches were complemented by screening of publications on websites of key organisations, and by citation tracking. Despite our extensive search, there might be a lack of representation from other regions outside the United States–Canada, the EU and the United Kingdom. The literature databases used in this review are international in scope, but are published in North America and Europe, and are known to be stronger in coverage of literature from those regions, so that, an unknown quantity of global literature not indexed in those databases was not scrutinised as part of this review.

In the same way, identification of other sources was biased towards websites and funders in the United States–Canada, the EU and the United Kingdom, due to lack of time and funding.

If this review is to be updated, it is possible to only run the electronic searches to obtain a quick actualisation of the recommendations. The records obtained searching other sources have strengthened the evidence found from the electronic searches, contributing more than half of the included studies, but they did not provide brand new information and searching other sources was a manual and time-consuming process. However, it could be worthwhile to directly seek updated records extracted from the Medical Research Council,^71^ European Medicines Agency,^57^ US Department of Health & Human Services^61,76,84^ and the Global Healthcare Data Science Community (Pharmaceutical Users Software Exchange – PhUSE).^23,24,58–60,67,69,70,72,73^

This review is exclusively gathering published recommendations/practices tailored specifically to clinical trials and it could not assess what researchers are actually using (but not reporting) for anonymising clinical trial data for sharing.

As this is a scoping review, there was no assessment of the quality of the evidence, therefore, we did not attempt to either explain how the included studies interpreted their local regulation on anonymisation, or to identify the existence of gaps in current practices from the obtained studies. The coding of the themes was a manual process and therefore subjective, however, a second reviewer sense-checked the coding and disagreements were mediated by a third reviewer which reduced the subjectivity of the findings.

## Conclusion

Currently, there is a strong demand for academic researchers to share their data more readily. In clinical trials, data can be shared more widely if they are anonymised, yet, we do not have standardised recommendations on how to do this. As time goes by there seems to be an emerging natural consensus on the definitions of pseudonymisation, de-identification and anonymisation.

The data manipulation techniques currently used are still simple, with an increasing amount of authors recommending a shift towards privacy models, such as k-anonymity. There are other privacy models but they are not routinely used in clinical trials, as they could be complex, time-consuming and not practical for clinical trials datasets (which are relatively small when compared against routine health data).

It is impossible to discuss anonymisation in clinical trials datasets without considering the way in which the data is going to be accessed. Controlled access is still the keystone for the release of clinical trial data.

Finally, an increasing number of authors agree that data should only be released if a threshold of acceptable risk is achieved, but there is not a clear definition of ‘acceptable’ as this is a very complex parameter that not only relies on the dataset but it is also embedded in a wider context out of scope for this review.

The studies identified during this review need to next be critically appraised to identify any gaps in the literature regarding anonymisation methods and data access approaches. Also, clear guidance on methods for quantifying the risk of re-identification need to be developed. This would allow for the creation of standardised worldwide recommendations for data sharing in clinical trials reflecting the growing consensus exhibited in the literature found during this review.

## Supplemental Material

sj-docx-2-ctj-10.1177_17407745221087469 – Supplemental material for Current recommendations/practices for anonymising data from clinical trials in order to make it available for sharing: A scoping review Click here for additional data file. Supplemental material, sj-docx-2-ctj-10.1177_17407745221087469 for Current recommendations/practices for anonymising data from clinical trials in order to make it available for sharing: A scoping review by Aryelly Rodriguez, Christopher Tuck, Marshall F Dozier, Stephanie C Lewis, Sandra Eldridge, Tracy Jackson, Alastair Murray and Christopher J Weir in Clinical Trials

sj-docx-3-ctj-10.1177_17407745221087469 – Supplemental material for Current recommendations/practices for anonymising data from clinical trials in order to make it available for sharing: A scoping review Click here for additional data file. Supplemental material, sj-docx-3-ctj-10.1177_17407745221087469 for Current recommendations/practices for anonymising data from clinical trials in order to make it available for sharing: A scoping review by Aryelly Rodriguez, Christopher Tuck, Marshall F Dozier, Stephanie C Lewis, Sandra Eldridge, Tracy Jackson, Alastair Murray and Christopher J Weir in Clinical Trials

sj-docx-4-ctj-10.1177_17407745221087469 – Supplemental material for Current recommendations/practices for anonymising data from clinical trials in order to make it available for sharing: A scoping review Click here for additional data file. Supplemental material, sj-docx-4-ctj-10.1177_17407745221087469 for Current recommendations/practices for anonymising data from clinical trials in order to make it available for sharing: A scoping review by Aryelly Rodriguez, Christopher Tuck, Marshall F Dozier, Stephanie C Lewis, Sandra Eldridge, Tracy Jackson, Alastair Murray and Christopher J Weir in Clinical Trials

sj-pdf-1-ctj-10.1177_17407745221087469 – Supplemental material for Current recommendations/practices for anonymising data from clinical trials in order to make it available for sharing: A scoping review Click here for additional data file. Supplemental material, sj-pdf-1-ctj-10.1177_17407745221087469 for Current recommendations/practices for anonymising data from clinical trials in order to make it available for sharing: A scoping review by Aryelly Rodriguez, Christopher Tuck, Marshall F Dozier, Stephanie C Lewis, Sandra Eldridge, Tracy Jackson, Alastair Murray and Christopher J Weir in Clinical Trials
