# Supplementary material for: Current recommendations/practices for anonymising data from clinical trials in order to make it available for sharing: A scoping review
Source: Clin Trials. 2022 Jun 22;19(4):452–63. doi: 10.1177/17407745221087469 (PMC9373195; doi:10.1177/17407745221087469)
Supplement: sj-docx-2-ctj-10.1177_17407745221087469 – Supplemental material for Current recommendations/practices for anonymising data from clinical trials in order to make it available for sharing: A scoping review [file sj-docx-2-ctj-10.1177_17407745221087469.docx]

| Appendix 2 – Search strategies for electronic databases/registries  11/02/2021 | | | | | | |
| --- | --- | --- | --- | --- | --- | --- |
| Study Recommendations/methods on anonymisation in clinical trials | | | | | | |
|  | **Catalogue** | | **Strategy** | | |  |
| Ovid MEDLINE(R) and In-Process & Other Non-Indexed Citations  Notes: lines 8 and 9 were added  to update the search | | 1.- (clinical adj2 (trial* or randomi* or research* or control*)).mp.  2.- (principle* or guid* or recomm*).mp. [mp=title, abstract, original title, name of substance word, subject heading word, floating sub-heading word, keyword heading word, protocol supplementary concept word, rare disease supplementary concept word, unique identifier, synonyms]  3.- (shar* or reus* or re-us* or access* or open).mp. [mp=title, abstract, original title, name of substance word, subject heading word, floating sub-heading word, keyword heading word, protocol supplementary concept word, rare disease supplementary concept word, unique identifier, synonyms]  4.- Data Anonymization/  5.- (de-identi* or deidenti* or anonym* or privacy or confidential*).mp. [mp=title, abstract, original title, name of substance word, subject heading word, floating sub-heading word, keyword heading word, protocol supplementary concept word, rare disease supplementary concept word, unique identifier, synonyms]  6.- 4 or 5  7.- 1 and 2 and 3 and 6  8.-(2019* or 2020* or 2021*).ed.  9.- 7 and 8 | | | | |
| Ovid Embase Classic + Embase  Notes: lines 12 and 13 were added  to update the search | | 1.- (clinical adj2 (trial* or randomi* or research* or control*)).mp.  2.- (principle* or guid* or recomm*).mp. [mp=title, abstract, heading word, drug trade name, original title, device manufacturer, drug manufacturer, device trade name, keyword, floating subheading word, candidate term word]  3.- (shar* or reus* or re-us* or access* or open).mp. [mp=title, abstract, heading word, drug trade name, original title, device manufacturer, drug manufacturer, device trade name, keyword, floating subheading word, candidate term word]  4.- Data Anonymization/  5.- (de-identi* or deidenti* or anonym* or privacy or confidential*).mp. [mp=title, abstract, heading word, drug trade name, original title, device manufacturer, drug manufacturer, device trade name, keyword, floating subheading word, candidate term word]  6.- 4 or 5  7.- 1 and 2 and 3 and 6  8.- anonymization/  9.- 5 or 8  10.- 1 and 2 and 3 and 9  11.- 7 and 10  12.- (2019* or 2020* or 2021*).em.  13.- 11 and 12 | | | | |
| Web of Science | | # 9 #8 AND #5 Indexes=SCI-EXPANDED, SSCI, A&HCI, CPCI-S, CPCI-SSH, BKCI-S, BKCI-SSH, ESCI, CCR-EXPANDED, IC Timespan=All years  # 8 LD=(1900-01-01/2019-02-10)  Indexes=SCI-EXPANDED, SSCI, A&HCI, CPCI-S, CPCI-SSH, BKCI-S, BKCI-SSH, ESCI, CCR-EXPANDED, IC Timespan=All years  # 7 #6 AND #5  Indexes=SCI-EXPANDED, SSCI, A&HCI, CPCI-S, CPCI-SSH, BKCI-S, BKCI-SSH, ESCI, CCR-EXPANDED, IC Timespan=All years  # 6 LD=(2019-02-11/2021-12-31)  Indexes=SCI-EXPANDED, SSCI, A&HCI, CPCI-S, CPCI-SSH, BKCI-S, BKCI-SSH, ESCI, CCR-EXPANDED, IC Timespan=All years  # 5 #1 AND #2 AND #3 AND #4  Indexes=SCI-EXPANDED, SSCI, A&HCI, CPCI-S, CPCI-SSH, BKCI-S, BKCI-SSH, ESCI, CCR-EXPANDED, IC Timespan=All years  # 4 TS=(de-identi* or deidenti* or anonym* or privacy or confidential*)  Indexes=SCI-EXPANDED, SSCI, A&HCI, CPCI-S, CPCI-SSH, BKCI-S, BKCI-SSH, ESCI, CCR-EXPANDED, IC Timespan=All years  # 3 TS=(shar* or reus* or re-us* or access* or open)  Indexes=SCI-EXPANDED, SSCI, A&HCI, CPCI-S, CPCI-SSH, BKCI-S, BKCI-SSH, ESCI, CCR-EXPANDED, IC Timespan=All years  # 2 TS=(principle* OR guid* OR recomm*)  Indexes=SCI-EXPANDED, SSCI, A&HCI, CPCI-S, CPCI-SSH, BKCI-S, BKCI-SSH, ESCI, CCR-EXPANDED, IC Timespan=All years  # 1 TS=(clinical NEAR/2 (trial* OR randomi* OR research* or control*) )  Indexes=SCI-EXPANDED, SSCI, A&HCI, CPCI-S, CPCI-SSH, BKCI-S, BKCI-SSH, ESCI, CCR-EXPANDED, IC Timespan=All years | | | | |
|  | | | | Page 1 of 1 |  | |
