# Supplementary material for: Current recommendations/practices for anonymising data from clinical trials in order to make it available for sharing: A scoping review
Source: Clin Trials. 2022 Jun 22;19(4):452–63. doi: 10.1177/17407745221087469 (PMC9373195; doi:10.1177/17407745221087469)
Supplement: sj-docx-3-ctj-10.1177_17407745221087469 – Supplemental material for Current recommendations/practices for anonymising data from clinical trials in order to make it available for sharing: A scoping review [file sj-docx-3-ctj-10.1177_17407745221087469.docx]

|  | | | | | | Appendix 3 – List of Included Records | | | | | | | | 16/01/2021 05:12 | | |  |
| --- | --- | --- | --- | --- | --- | --- | --- | --- | --- | --- | --- | --- | --- | --- | --- | --- | --- |
| Study Recommendations/methods on anonymisation in clinical trials | | | | | | | | | | | | | | | | |  |
|  | **Study  id** | | **Main Study  name** | **Articles in Study** | **Nodes coded** | | | **Article classification** | **First Authors** | | | **Country** | **Article Complete title** | | **year** |  | |
| 1 | | 1g_ANMRD | | 1 | 7 | | Other  Sources | | Australian National Medical Research Data Storage Facility ^1^ | | | Australia | Anonymisation | | 2016 | | |
| 2 | | 1g_ASTHMA UK | | 1 | 6 | | Other  Sources | | Asthma UK Centre for Applied Research ^2^ | | | UK | ASTHMA UK policy data sharing - Introduction to sharing individual participant data | | 2015 | | |
| 3 | | 1g_E Emam | | 1 | 11 | | Other  Sources | | El Emam, Khaled ^3^ | | | Canada | Concepts And methods for de-identifying clinical trial data | | 2014 | | |
| 4 | | 1g_Ebner | | 1 | 7 | | Other  Sources | | Ebner, Hubert ^4^ | | | EU | Piloting the European unified patient identity management (EUPID) concept to facilitate secondary use of neuroblastoma data from Clinical Trials and Biobanking | | 2016 | | |
|  |  |  |  |  |  |  |  |  |  |  |  |  |  |  |  |  |  |
| 5 | | 1g_EMA | | 1 | 13 | | Other  Sources | | European Medicines Agency (EMA) ^5^ | | | EU | Data anonymisation - a key enabler for clinical data sharing Workshop report | | 2018 | | |
| 6 | | 1g_healthdata | | 1 | 7 | | Other  Sources | | The Expert Panel on Timely Access to Health and Social Data for Health Research and Health System Innovation ^6^ | | | Canada | Accessing Health and Health-Related Data in Canada | | 2015 | | |
| 7 | | 1g_hhs | | 3 | 4 | | Other  Sources | | Food Drug Administration ^7^ | | | US | HSS - Availability of masked and de-identified non-summary safety and efficacy data; request for comments | | 2013 | | |
|  |  |  |  |  | 5 | | Other  Sources | | National Institutes of Health (NIH) ^8^ | | | US | HSS - Clinical research and the HIPAA privacy rule | | 2012 | | |
|  |  |  |  |  | 12 | | Other  Sources | | U.S. Department of Health & Human Services (HHS) ^9^ | | | US | HSS - Guidance regarding methods for de-identification of protected health information in accordance with the Health Insurance Portability and Accountability Act (HIPAA) Privacy Rule | | 2012 | | |
| 8 | | 1g_Hollis | | 1 | 7 | | Other  Sources | | Hollis, Sally ^10^ | | | EU | Best practice for analysis of shared clinical trial data | | 2016 | | |
| 9 | | 1g_Hughes | | 1 | 9 | | Other  Sources | | Hughes, Sara ^11^ | | | UK | Preparing individual patient data from clinical trials for sharing: the GlaxoSmithKline approach | | 2014 | | |
| 10 | | 1g_Huser | | 1 | 8 | | Other  Sources | | Huser, Vojtech ^12^ | | | US | Data sharing platforms for de-identified data from human clinical trials | | 2018 | | |
| 11 | | 1g_IoM | | 1 | 21 | | Other  Sources | | IOM (Institute of Medicine) ^13^ | | | US | Sharing Clinical Trial Data: Maximizing Benefits, Minimizing Risk | | 2015 | | |
| 12 | | 1g_IPPC | | 1 | 9 | | Other  Sources | | International Pharmaceutical Privacy Consortium ^14^ | | | US-EU- UK | IPPC White Paper on Anonymisation of Clinical Trial  Datasets. | | 2014 | | |
| 13 | | 1g_Jonas | | 1 | 3 | | Other  Sources | | Jonas, Stephan ^15^ | | | EU | Privacy-Preserving Record Grouping and Consent Management Based on a Public-Private Key Signature Scheme: Theoretical Analysis and Feasibility Study | | 2019 | | |
| 14 | | 1g_Miller | | 1 | 4 | | Other  Sources | | Miller, James D ^16^ | | | US | Sharing clinical research data in the United States under the health insurance portability and accountability act and the privacy rule | | 2010 | | |
| 15 | | 1g_MRC | | 1 | 5 | | Other  Sources | | Medical Research Council (MRC), ^17^ | | | UK | GDPR Guidance note 5: Identifiability, anonymisation and pseudonymisation | | 2019 | | |
| 16 | | 1g_Nelson | | 1 | 13 | | Other  Sources | | Nelson, Gregory S. ^18^ | | | US | Practical Implications of Sharing Data: A Primer on Data Privacy, Anonymization, and De-Identification | | 2015 | | |
| 17 | | 1g_NIH | | 1 | 6 | | Other  Sources | | National Institutes of Health (NIH) ^19^ | | | US | NIH data sharing policy and implementation guidance | | 2003 | | |
| 18 | | 1g_Pfizer | | 1 | 5 | | Other  Sources | | Pfizer ^20^ | | | EU | Clinical Trial Data Access -Policy Document 01312014 | | 2014 | | |
| 19 | | 1g_PhUSE | | 10 | 10 | | Other  Sources | | Ferran, Jean-Marc ^21^ | | | EU | PhUSE De-Identification Working Group: Providing De-Identification Standards to CDISC Data Models | | 2015 | | |
|  |  |  |  |  | 9 | | Other  Sources | | Ferran, Jean-Marc ^22^ | | | EU | PhUSE De-Identification Working Group: Providing  De-Identification Standards to CDISC Data  Models - DS10 - Old version of DH01 | | 2015 | | |
|  |  |  |  |  | 4 | | Other  Sources | | Ferran, Jean-Marc ^23^ | | | EU | PhUSE - De-Identification Standards for CDISC Data  Models - PhUSE, Data Transparency Working Group Lead | | 2017 | | |
|  |  |  |  |  | 6 | | Other  Sources | | Iversen, Jørgen Mangor ^24^ | | | EU | PhUSE - Data De-Identification Made Simple | | 2016 | | |
|  |  |  |  |  | 11 | | Other  Sources | | Kniola, Lukasz ^25^ | | | EU | PhUSE - Data Anonymisation and Risk Assessment Automation | | 2020 | | |
|  |  |  |  |  | 7 | | Other  Sources | | Lyathakula, Santhosh ^26^ | | | EU | PhUSE - Data Anonymization Providing clinical trial data to outside researchers | | 2015 | | |
|  |  |  |  |  | 7 | | Other  Sources | | Meeh, Sherry ^27^ | | | EU | PhUSE Data De-identification Standard for CDSIC  SDTM IG 3.2, and EMA Policy 0070 | | 2016 | | |
|  |  |  |  |  | 4 | | Other  Sources | | Meeh, Sherry ^28^ | | | EU | PhUSE Data De-identification Standard for CDSIC  ADaM 2.1 IG 1.0, and Updates for SDTM IG 3.2 | | 2017 | | |
|  |  |  |  |  | 3 | | Other  Sources | | PhUSE ^29^ | | | EU | PhUSE DeID Standard - SDTM 3.2 - Appendix 1 - Date Offsetting - v1.91[2] | | 2015 | | |
|  |  |  |  |  | 6 | | Other  Sources | | PhUSE ^30^ | | | EU | PhUSE Data De-Identification Standard for SDTM 3.2 -appendix 2-low frequencies-v10-19387 | | 2015 | | |
| 20 | | 1g_Shostak | | 1 | 10 | | Other  Sources | | Shostak, Jack ^31^ | | | US | De-Identification of clinical trials data demystified | | 2006 | | |
|  | | | | | | | | | | Page 1 of 2 |  | | | | | |  |

|  | | | | | | | Appendix 3 – List of Included Records | | | | | | | | | 16/01/2021 05:12 | | |  |
| --- | --- | --- | --- | --- | --- | --- | --- | --- | --- | --- | --- | --- | --- | --- | --- | --- | --- | --- | --- |
|  | **Study  id** | | **Main Study  name** | **Articles in Study** | **Nodes coded** | | | **Article classification** | **First Authors** | | | | **Country** | | **Article Complete title** | | **year** |  | |
| 21 | | 1g_sponsor statements | | 3 | 7 | Other  Sources | | | | Clinical Study Data Request (CSDR) ^32^ | | | | EU | CSDR - Anonymisation of Clinical Trial Datasets | | 2015 | | |
|  |  |  |  |  | 9 | Other  Sources | | | | Clinical Study Data Request (CSDR) ^33^ | | | | EU | CSDR - Anonymisation of Clinical Trial Datasets – Eli Lilly  and Company | | 2015 | | |
|  |  |  |  |  | 8 | Other  Sources | | | | Clinical Study Data Request (CSDR) ^34^ | | | | EU | CSDR - Anonymisation of Clinical Trial Datasets - Eisai | | 2015 | | |
| 22 | | 1g_transcelerate | | 2 | 14 | Other  Sources | | | | TransCelerate BioPharma Inc ^35^ | | | | US-EU- UK | TransCelerate-Data de-identification and anonymization of individual patient data in clinical studies | | 2013 | | |
|  |  |  |  |  | 13 | Other  Sources | | | | TransCelerate BioPharma Inc ^36^ | | | | US-EU- UK | TransCelerate-Anonymization of Individual Patient Data in Clinical Studies–A Model Approach | | 2015 | | |
| 23 | | 1g_Walker | | 1 | 9 | Other  Sources | | | | Walker, Neil ^37^ | | | | UK | All or Nothing: The False Promise of Anonymity | | 2017 | | |
| 24 | | 2g_ANDS | | 1 | 11 | Other  Sources | | | | Olesen, Sarah^38^ | | | | Australia | Publishing and Sharing Sensitive Data | | 2011 | | |
| 25 | | Atzor | | 1 | 11 | Electronic  Search | | | | Atzor, S. ^39^ | | | | EU | Clinical trial data sharing: From principles to practical implementation - An industry model | | 2014 | | |
| 26 | | Demotes-Mainard | | 1 | 8 | Electronic  Search | | | | Demotes-Mainard, J. ^40^ | | | | EU | How the new European data protection regulation  affects clinical research and recommendations? | | 2019 | | |
| 27 | | El Emam_2008 | | 1 | 7 | Electronic  Search | | | | El Emam, K. ^41^ | | | | Canada | Protecting privacy using k-anonymity | | 2008 | | |
| 28 | | El Emam_2015 | | 1 | 13 | Electronic  Search | | | | El Emam, K. ^42^ | | | | Canada | Anonymising and sharing individual patient data | | 2015 | | |
| 29 | | Hrynaszkiewicz | | 1 | 12 | Electronic  Search | | | | Hrynaszkiewicz, I. ^43^ | | | | UK | Preparing raw clinical data for publication: guidance  for journal editors, authors, and peer reviewers | | 2010 | | |
| 30 | | Keerie | | 1 | 12 | Electronic  Search | | | | Keerie, C ^44^. | | | | UK | Data sharing in clinical trials - practical guidance on anonymising trial datasets | | 2018 | | |
| 31 | | Lee | | 1 | 9 | Electronic  Search | | | | Lee, J. ^45^ | | | | Korea | Design of a human-centric de-identification framework  for utilizing various clinical research data | | 2018 | | |
| 32 | | Malin | | 1 | 8 | Electronic  Search | | | | Malin, B. ^46^ | | | | US | Technical and Policy Approaches to Balancing Patient  Privacy and Data Sharing in Clinical and Translational Research | | 2010 | | |
| 33 | | Morse | | 1 | 5 | Electronic  Search | | | | Morse, R ^47^ | | | | US | Web-browser encryption of personal health information | | 2011 | | |
| 34 | | Nasseh | | 1 | 5 | Electronic  Search | | | | Nasseh, D ^48^ | | | | EU | Matching study to registry data: maintaining data privacy in  a study on family based colorectal cancer | | 2014 | | |
| 35 | | Nitzlnader | | 1 | 7 | Electronic  Search | | | | Nitzlnader, M ^49^ | | | | EU | Patient identity management for secondary use of  biomedical research data in a distributed computing environment | | 2014 | | |
| 36 | | Noumeir | | 1 | 7 | Electronic  Search | | | | Noumeir, R. ^50^ | | | | Canada | Pseudonymization of radiology data for research purposes | | 2007 | | |
| 37 | | Ohmann | | 2 | 8 | Electronic  Search | | | | Ohmann, C ^51^ | | | | EU | Sharing and reuse of individual participant data from clinical trials: principles and recommendations | | 2017 | | |
|  |  |  |  |  | 6 | Electronic  Search | | | | Ohmann, C - Supplement ^51^ | | | | EU | Sharing and reuse of individual participant data from  clinical trials: principles and recommendations | | 2017 | | |
| 38 | | Schell | | 1 | 10 | Electronic  Search | | | | Schell, S ^52^ | | | | US | Creation of clinical research databases in the 21st century:  a practical algorithm for HIPAA Compliance | | 2006 | | |
| 39 | | Sudlow | | 1 | 7 | Electronic  Search | | | | Sudlow, R. ^53^ | | | | UK | EFSPI/PSI working group on data sharing: accessing and working with pharmaceutical clinical trial patient level datasets–a primer for academic researchers | | 2016 | | |
| 40 | | Tuck | | 2 | 4 | Electronic  Search | | | | Tuck, C ^54^ | | | | UK | Data sharing in clinical trials - practical guidance on anonymising trial datasets - Oral Presentation | | 2015 | | |
|  |  |  |  |  | 8 | Electronic  Search | | | | Tuck, C ^54^ | | | | UK | Presentation | | 2015 | | |
| 41 | | Tucker | | 1 | 15 | Electronic  Search | | | | Tucker, K. ^55^ | | | | UK | Protecting patient privacy when sharing patient-level data from clinical trials | | 2016 | | |
| 42 | | Tudur-Smith 2015-2017 | | 3 | 11 | Electronic  Search | | | | Tudur Smith, C ^56^ | | | | UK | How should individual participant data (IPD) from publicly funded clinical trials be shared? | | 2015 | | |
|  |  |  |  |  | 5 | Electronic  Search | | | | Tudur Smith, C ^57^ | | | | UK | Good practice principles for sharing individual participant data from publicly funded clinical trials | | 2015 | | |
|  |  |  |  |  | 8 | Electronic  Search | | | | Tudur Smith, C ^58^ | | | | UK | Resource implications of preparing individual participant  data from a clinical trial to share with external researchers | | 2017 | | |
| 43 | | Wallace | | 1 | 3 | Electronic  Search | | | | Wallace, S. ^59^ | | | | UK | Protecting personal data in epidemiological research: DataSHIELD and UK law | | 2014 | | |
|  | | | | | | | | | | | Page 2 of 2 |  | | | | | | |  |

**Reference List**

1. Australian National Medical Research Data Storage Facility. Anonymisation. circa 2016.

2. Asthma UK Centre for Applied Research. ASTHMA UK policy data sharing - Introduction to sharing individual participant data. Version 2 ed. circa 2015, p. 2.

3. El Emam K and Malin B. Concepts And methods for de-identifying clinical trial data. *Paper commissioned by the Committee on Strategies for Responsible Sharing of Clinical Trial Data*. 2014.

4. Ebner H, Hayn D, Falgenhauer M, et al. Piloting the European unified patient identity management (EUPID) concept to facilitate secondary use of neuroblastoma data from Clinical Trials and Biobanking. *Health Informatics Meets EHealth: Predictive Modeling in Healthcare–From Prediction to Prevention Proceedings of the 10th EHealth2016 Conference*. IOS Press, 2016, p. 31-8.

5. European Medicines Agency (EMA). Data anonymisation - a key enabler for clinical data sharing Workshop report. In: Agency EM, (ed.). *EMA/796532/2018*. London2018

6. The Expert Panel on Timely Access to Health and Social Data for Health Research and Health System Innovation. Accessing Health and Health-Related Data in Canada. Printed in Ottawa, Canada: Council of Canadian Academies Ottawa, Ontario, Canada, 2015.

7. Food Drug Administration. HHS - Availability of masked and de-identified non-summary safety and efficacy data; request for comments. *Federal Register Available:* [*https://wwwfederalregistergov/articles/2013/06/04/2013-13083/availability-of-masked-and-de-identified-non-summary-safety-and-efficacy-data-request-for-comments*](https://wwwfederalregistergov/articles/2013/06/04/2013-13083/availability-of-masked-and-de-identified-non-summary-safety-and-efficacy-data-request-for-comments). 2013.

8. National Institutes of Health (NIH). HHS - Clinical research and the HIPAA privacy rule. *Retreived from* [*http://privacyruleandresearch*](http://privacyruleandresearch) *nih gov/clin_research asp*. 2004.

9. U.S. Department of Health & Human Services (HHS). HHS - Guidance regarding methods for de-identification of protected health information in accordance with the Health Insurance Portability and Accountability Act (HIPAA) Privacy Rule. *US Department of Health and Human Services, Washington, DC) Available at :*[*https://wwwhhsgov/hipaa/for-professionals/privacy/special-topics/de-identification/indexhtml*](https://wwwhhsgov/hipaa/for-professionals/privacy/special-topics/de-identification/indexhtml). 2012; 26.

10. Hollis S, Fletcher C, Lynn F, et al. Best practice for analysis of shared clinical trial data. *BMC medical research methodology*. 2016; 16 Suppl 1: 76.

11. Hughes S, Wells K, McSorley P and Freeman A. Preparing individual patient data from clinical trials for sharing: the GlaxoSmithKline approach. *Pharmaceutical Statistics*. 2014; 13: 179-83.

12. Huser V and Shmueli-Blumberg D. Data sharing platforms for de-identified data from human clinical trials. *Clinical Trials*. 2018; 15: 413-23.

13. IOM (Institute of Medicine). *Sharing Clinical Trial Data: Maximizing Benefits, Minimizing Risk*. Washington (DC): National Academies Press, 2015.

14. International Pharmaceutical Privacy Consortium. IPPC White Paper on Anonymisation of Clinical Trial Datasets. *Online:* [*http://pharmaprivacyorg/activities/ippc-white-paper-on-anonymisation-of-clinical-trial-data-sets*](http://pharmaprivacyorg/activities/ippc-white-paper-on-anonymisation-of-clinical-trial-data-sets). 2014.

15. Jonas S, Siewert S and Spreckelsen C. Privacy-Preserving Record Grouping and Consent Management Based on a Public-Private Key Signature Scheme: Theoretical Analysis and Feasibility Study. *Journal of medical Internet research*. 2019; 21: e12300.

16. Miller JD. Sharing clinical research data in the United States under the health insurance portability and accountability act and the privacy rule. *Trials*. 2010; 11: 112.

17. Medical Research Council (MRC). GDPR Guidance note 5: Identifiability, anonymisation and pseudonymisation. 2019.

18. Nelson GS. Practical Implications of Sharing Data: A Primer on Data Privacy, Anonymization, and De-Identification. In: SAS, (ed.). *SAS GLOBAL FORUM Procedings 2015*. 2015, p. 1-23.

19. National Institutes of Health (NIH). NIH data sharing policy and implementation guidance. *Retrieved June*. 2003; 18: 2009.

20. Pfizer. Clinical Trial Data Access -Policy Document 01312014. 2014.

21. Ferran J-M, El Emam K, Nolan S, Grimm B and De Donder N. PhUSE De-Identification Working Group: Providing De-Identification Standards to CDISC Data Models. *PhUSE*. 2015.

22. Ferran J-M and Lanoue J. PhUSE De-Identification Working Group: Providing De-Identification Standards to CDISC Data Models - DS10 - Old version of DH01. *PharmaSUG 2015 - Paper DS10 - Old version of DH01*. 2015.

23. Ferran J-M. PhUSE - De-Identification Standards for CDISC Data Models - PhUSE, Data Transparency Working Group Lead. *4th International Clinical Trials Methodology Conference (ICTMC)* Liverpool2017.

24. Iversen JM. PhUSE - Data De-Identification Made Simple. *PHUSE - LEO Pharma A/S, Ballerup, Denmark*. 2016.

25. Kniola L, Hughes A, Paczewska-Sosnowska A, et al. PhUSE - Data Anonymisation and Risk Assessment Automation. *PhUSE*. 2020; 1.0.

26. Lyathakula S. PhUSE - Data Anonymization Providing clinical trial data to outside researchers. In: NOVARTIS, (ed.). *PhUSE Single Day Event (SDE)*  Mumbai 2015.

27. Meeh S. PhUSE Data De-identification Standard for CDSIC SDTM IG 3.2, and EMA Policy 0070. In: Janssen IDAaR-, (ed.). . 2016.

28. Meeh S. PhUSE Data De-identification Standard for CDSIC ADaM 2.1 IG 1.0, and Updates for SDTM IG 3.2. 2017.

29. PhUSE. PhUSE DeID Standard - SDTM 3.2 - Appendix 1 - Date Offsetting - v1.91[2]. In: PhUSE, (ed.). PhUSE 2015.

30. PhUSE. PhUSE Data De-Identification Standard for SDTM 3.2 -appendix 2-low frequencies-v10-19387. 2015.

31. Shostak J. De-Identification of clinical trials data demystified. *SAS Users Group*. 2006.

32. Clinical Study Data Request (CSDR). CSDR - Anonymisation of Clinical Trial Datasets. circa 2015.

33. Clinical Study Data Request (CSDR) EL. CSDR - Anonymisation of Clinical Trial Datasets – Eli Lilly and Company. circa 2015.

34. Clinical Study Data Request (CSDR) and Eisai. CSDR - Anonymisation of Clinical Trial Datasets - Eisai circa 2015.

35. TransCelerate BioPharma Inc. TransCelerate-Anonymization of Individual Patient Data in Clinical Studies–A Model Approach. De-identification, Transcelerate-Data, 2015.

36. TransCelerate BioPharma Inc. TransCelerate-Data de-identification and anonymization of individual patient data in clinical studies. *TransCelerate - Clinical Data Transparency Initiative*. 2016.

37. Walker N. All or Nothing: The False Promise of Anonymity. *Data Science Journal*. 2017; 16.

38. Olesen S. Publishing and Sharing Sensitive Data. *Australian National Data Service*. 2011.

39. Atzor S, Sorof J, Kelman A, et al. Clinical trial data sharing: From principles to practical implementation - An industry model. *Regulatory Rapporteur*. 2014; 11: 4-7.

40. Demotes-Mainard J, Cornu C, Guerin A, et al. How the new European data protection regulation affects clinical research and recommendations? *Therapie*. 2019.

41. El Emam K and Dankar FK. Protecting privacy using k-anonymity. *Journal of the American Medical Informatics Association*. 2008; 15: 627-37.

42. El Emam K, Rodgers S and Malin B. Anonymising and sharing individual patient data. *BMJ*. 2015; 350: h1139.

43. Hrynaszkiewicz I, Norton ML, Vickers AJ and Altman DG. Preparing raw clinical data for publication: guidance for journal editors, authors, and peer reviewers. *Trials*. 2010; 11.

44. Keerie C, Tuck C, Milne G, Eldridge S, Wright N and Lewis SC. Data sharing in clinical trials - practical guidance on anonymising trial datasets. *Trials*. 2018; 19: 25.

45. Lee J, Jung J, Park P, Chung S and Cha H. Design of a human-centric de-identification framework for utilizing various clinical research data. *Human-Centric Computing and Information Sciences*. 2018; 8.

46. Malin B, Karp D and Scheuermann RH. Technical and Policy Approaches to Balancing Patient Privacy and Data Sharing in Clinical and Translational Research. *Journal of Investigative Medicine*. 2010; 58: 11-8.

47. Morse RE, Nadkarni P, Schoenfeld DA and Finkelstein DM. Web-browser encryption of personal health information. *BMC medical informatics and decision making*. 2011; 11: 70.

48. Nasseh D, Engel J, Mansmann U, Tretter W and Stausberg J. Matching study to registry data: maintaining data privacy in a study on family based colorectal cancer. *e-Health – For Continuity of Care*. 2014.

49. Nitzlnader M and Schreier G. Patient identity management for secondary use of biomedical research data in a distributed computing environment. *eHealth*. 2014, p. 211-8.

50. Noumeir R, Lemay A and Lina JM. Pseudonymization of radiology data for research purposes. *Journal of Digital Imaging*. 2007; 20: 284-95.

51. Ohmann C, Banzi R, Canham S, et al. Sharing and reuse of individual participant data from clinical trials: principles and recommendations. *BMJ Open*. 2017; 7.

52. Schell SR. Creation of clinical research databases in the 21st century: a practical algorithm for HIPAA Compliance. *Surgical Infections*. 2006; 7: 37-44.

53. Sudlow R, Branson J, Friede T, Morgan D and Whately-Smith C. EFSPI/PSI working group on data sharing: accessing and working with pharmaceutical clinical trial patient level datasets–a primer for academic researchers. *BMC medical research methodology*. 2016; 16: 73.

54. Tuck C, Lewis S, Milne G, Eldridge S and Wright N. Data sharing in clinical trials - practical guidance on anonymising trial datasets - Oral Presentation. *Trials*. 2015; 16.

55. Tucker K, Branson J, Dilleen M, et al. Protecting patient privacy when sharing patient-level data from clinical trials. *BMC Medical Research Methodology*. 2016; 16 Suppl 1: 77.

56. Tudur Smith C, Hopkins C, Sydes MR, et al. How should individual participant data (IPD) from publicly funded clinical trials be shared? *BMC Medicine*. 2015; 13: 298.

57. Tudur Smith C, Hopkins C, Sydes MR, et al. Good practice principles for sharing individual participant data from publicly funded clinical trials Version 1 ed.: Medical Research Council - Hubs for Trials Methodology Research, 2015.

58. Tudur Smith C, Nevitt S, Appelbe D, et al. Resource implications of preparing individual participant data from a clinical trial to share with external researchers. *Trials*. 2017; 18: 319.

59. Wallace SE, Gaye A, Shoush O and Burton PR. Protecting personal data in epidemiological research: DataSHIELD and UK law. *Public Health Genomics*. 2014; 17: 149-57.
