# Supplementary material for: Current recommendations/practices for anonymising data from clinical trials in order to make it available for sharing: A scoping review
Source: Clin Trials. 2022 Jun 22;19(4):452–63. doi: 10.1177/17407745221087469 (PMC9373195; doi:10.1177/17407745221087469)
Supplement: sj-docx-4-ctj-10.1177_17407745221087469 – Supplemental material for Current recommendations/practices for anonymising data from clinical trials in order to make it available for sharing: A scoping review [file sj-docx-4-ctj-10.1177_17407745221087469.docx]

| Online Supplemental Figure 1 – PRISMA flow diagram |
| --- |
| 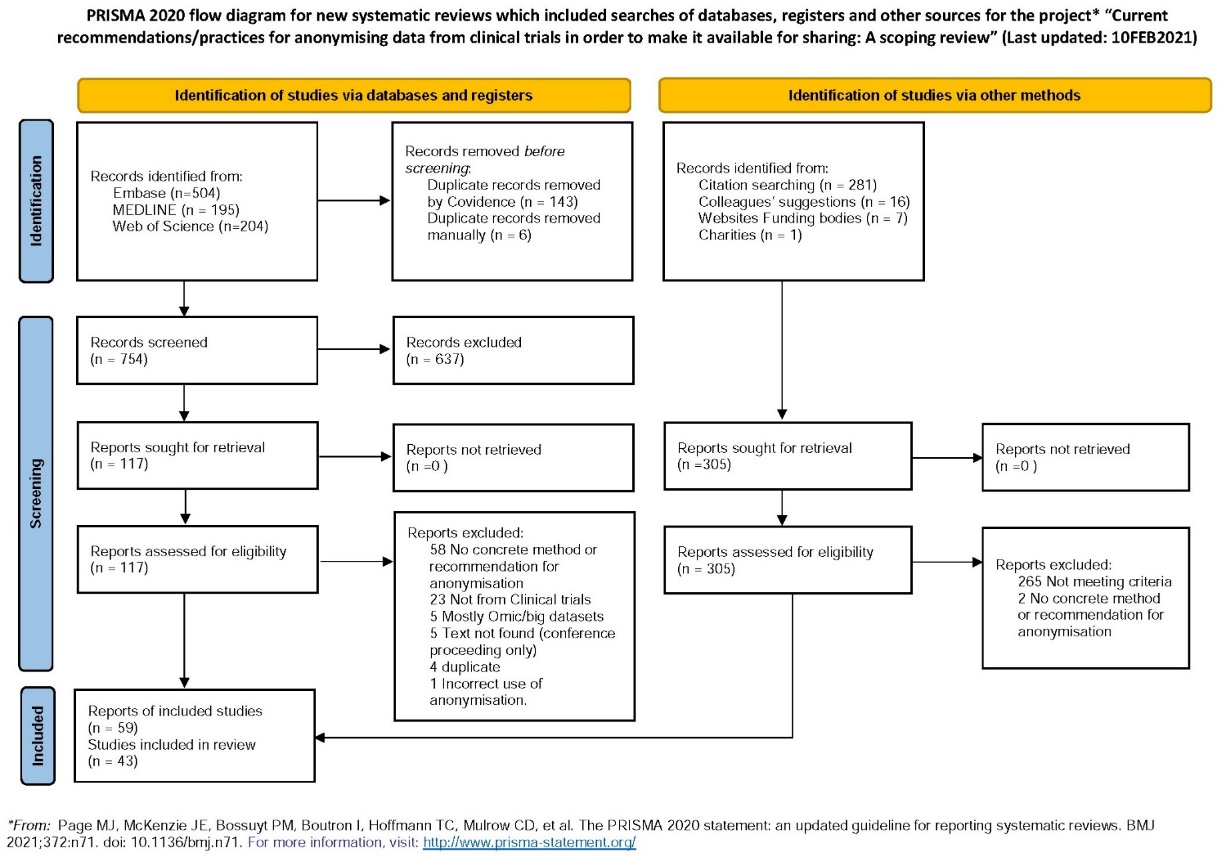 |

| Online Supplemental Figure 2 – Studies over time by Source and Country/Region | |
| --- | --- |
| 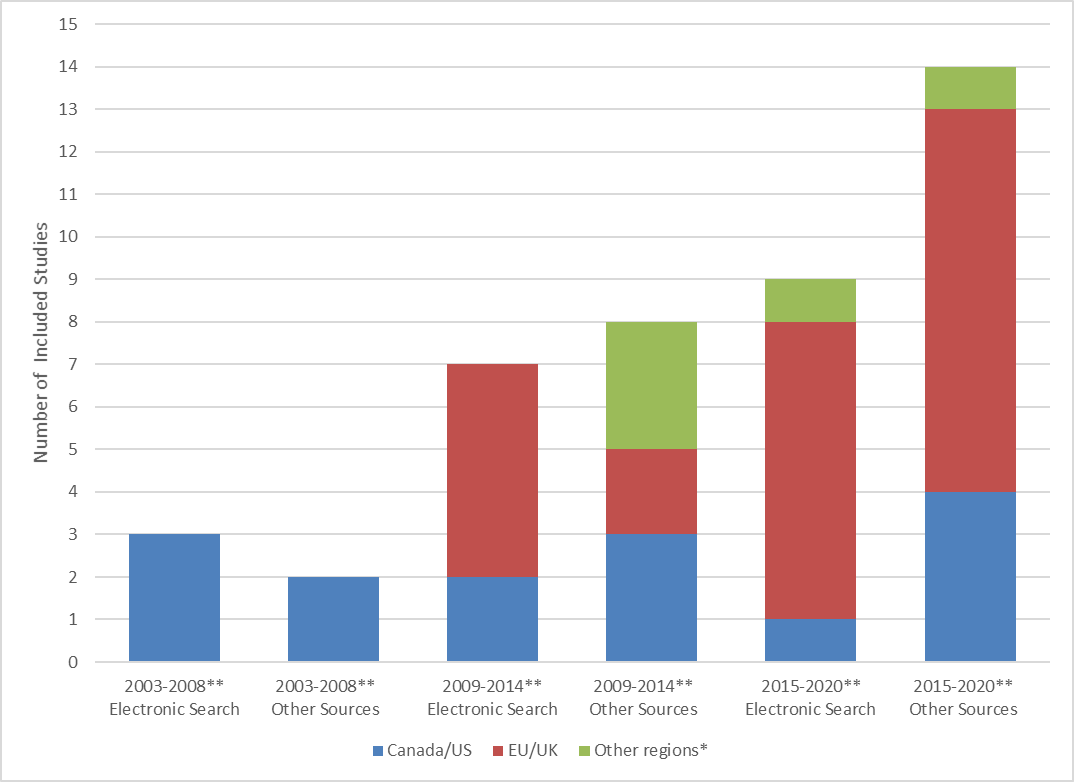 | |
| Notes: | *Consisting of Australia, US-EU-UK and South Korea.  **Where applicable the oldest record in the included study determined the overall study date. |

| Online Supplemental Figure 3 NVivo® Word Cloud | |
| --- | --- |
| 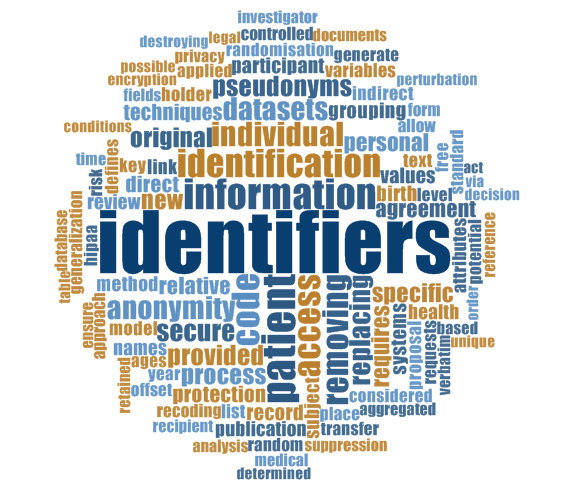 | |
| Notes: | Created 01 September 2019. |

| Online Supplemental Table 1 – Identified themes | |
| --- | --- |
| Theme description | Theme Id |
| When authors provided a clear definition of anonymisation, de-identification and pseudonymisation. | Text coded to 1, 2 or 3 respectively |
| If authors mentioned and described the removal of “The Health Insurance Portability and Accountability Act of 1996” (HIPAA) or Hrynaszkiewics identifiers. | Text coded to 2.1 or 2.2 respectively |
| If authors described manipulation of data in general. | Text coded to 4 |
| If authors explained with further detail the manipulation of data used (e.g. Perturbation, Recalculation, Recoding, Suppression or Remove superfluous data). | Text coded to 4.1 to 4.5, categories were added as needed |
| If authors mentioned and described a privacy model ^88-90^ (i.e. the dataset must satisfy certain conditions to keep the re-identification risk at or below an acceptable level. Privacy models usually depend on an algorithm that uses variables on the dataset to determine how much re-identification risk is present.). | Text coded to 4 |
| If authors explained with further detail the privacy model used (e.g. k-anonymity,^88^ l-diversity,^89^ differential privacy.^90^). | Text coded to 5.1, categories were added as needed |
| If authors explained, advocated or provided examples of “controlled access” to anonymised/de-identified datasets (e.g. the use of data sharing agreements, the location of data behind a secure access barrier, the identification and vetoing of secondary researchers (e.g. checking requesters are bona fide researchers with a valid research question). | Text coded to 6 |
| If authors explained with further detail the control access method (e.g. black box where data cannot be seen but the variables are available to make queries and results can be generated, end to end encryption, use of safe haven, or split location for datasets). | Text coded to 6.1 to 6.4, categories were added as needed |
| If authors explained, advocated or provided examples of “open access” to datasets, where minimal amount of (or non-existent) requirements for allowing access to the data set by secondary researchers. (e.g. free access to the dataset via a website.^91^ | Text coded to 7 |
| If authors explained, advocated or provided examples of central repository (e.g. a web page) in which researchers could deposit their dataset regardless of their affiliation. | Text coded to 8 |
| If authors explained that data could be released using expert determination in which a qualified person could deem the risk of re-identification to be small enough to allow such release. | Text coded to 9 |
| If authors explained that other information/documents should be provided with the clinical trial dataset (e.g. annotated case report forms (CRFs), statistical analysis plans (SAPs), study protocols, data dictionaries, anonymisation/de-identification techniques used for generating the released dataset, Clinical Summary Report (CSR), data sharing plans). | Text coded to 10 |
| If authors explained that a re-identification risk assessment should be carried out on the anonymised/de-identified datasets. | Text coded to 11 |
