# Supplementary material for: Current recommendations/practices for anonymising data from clinical trials in order to make it available for sharing: A scoping review
Source: Clin Trials. 2022 Jun 22;19(4):452–63. doi: 10.1177/17407745221087469 (PMC9373195; doi:10.1177/17407745221087469)
Supplement: sj-pdf-1-ctj-10.1177_17407745221087469 – Supplemental material for Current recommendations/practices for anonymising data from clinical trials in order to make it available for sharing: A scoping review [file sj-pdf-1-ctj-10.1177_17407745221087469.pdf]

Current recommendations/practices for anonymising data from clinical trials in order to make it available for sharing: Protocol for a scoping review

Rodriguez A[1], Tuck C.[1], Dozier MF[2], Lewis SC[1], Eldridge S[3], Weir CJ[1]

[1]Edinburgh Clinical Trials Unit, Usher Institute of Population Health Sciences and Informatics, the University of Edinburgh

[2]Library & University Collections, Information Services, the University of Edinburgh

[3]Pragmatic Clinical Trials Unit, Blizard Institute, Barts and the London School of Medicine and Dentistry, Queen Mary University of London

Correspondence:
Ms Aryelly Rodriguez
Edinburgh Clinical Trials Unit, the University of Edinburgh
Level 2, Nine Edinburgh BioQuarter,
9 Little France Road, Edinburgh, EH16 4UX
Emails: aryelly.rodriguez@ed.ac.uk, chris.tuck@ed.ac.uk, marshall.dozier@ed.ac.uk steff.lewis@ed.ac.uk, christopher.weir@ed.ac.uk, s.eldridge@qmul.ac.uk

Protocol registration

The protocol will not be registered with the International Prospective Register of Systematic Reviews (PROSPERO) as the proposed systematic review does not meet the PROSPERO inclusion criteria.

Current recommendations/practices for anonymising data from clinical trials in order to make it available for sharing: Protocol for a scoping review

Abstract

There are increasing pressures for anonymised datasets from clinical trials to be shared across the scientific community. There are various sets of recommendations on how to perform anonymisation prior to sharing clinical trial data. We aim to systematically identify, describe and synthesise these recommendations. We will systematically search literature databases and websites of key organisations in the field. Any publication reporting recommendations on anonymisation to enable data sharing in clinical trials will be included. Two reviewers will independently screen titles, abstracts and full text for eligibility. One reviewer will extract data from included papers which will then be sense checked by a second reviewer. Results will be summarised by narrative review. This scoping review will provide information about existing recommendations for anonymising clinical trial datasets in order to make them available for sharing and it will inform (if applicable) the development of new recommendations.

Key Words: Clinical Trials | Systematic Review | Data Anonymization | Patient Identification Systems | Personally Identifiable Information | Datasets | Data Curation | Guidelines

Current recommendations/practices for anonymising data from clinical trials in order to make it available for sharing: Protocol for a scoping review

Background

When academic-led clinical trials are completed, their results are usually released to the public and wider scientific community in scientific journals or clinical trials registries. However, generally, there are considerable amounts of data that are not analysed as part of the final report [1]. In addition, it is often useful to perform meta-analyses across several trials and using the individual patient data from each trial adds to the quality of such analyses [2], for instance by allowing full investigation of subgroup effects. Clinical trials are complex, time-consuming and costly, and it is wasteful not to use data fully [3]. There is now a drive, particularly from publishers and funders, to encourage the release of relevant anonymised trial data sets [4].

Clinical trial datasets contain personal health information on the trial participants. It is imperative that data sharing does not disclose personal data to anyone who falls outside the original group to whom the trial participants consented to disclose their data. Anonymising the trial dataset fulfils this requirement. However, the anonymisation process removes information from the data, and if not done properly, the original trial analyses could not be reproduced, which in turn will limit the data's usability for further research [5]. Anonymisation is complex, and there are many possible ways of performing it.

The drive to share data more widely has generated various sets of recommendations to enable sharing [4, 6-9]. Embedded within these, there is a variety of recommendations on how to anonymise a dataset.

Why it is important to do this review

To our knowledge, there are no reviews of the methods and/or recommendations for the process of generating anonymised clinical trial datasets[1]. To understand and bring together

[1] A quick search was executed on the 07JAN2019 on Google Scholar with "literature" "review" "anonymization" "methods" "clinical trials" and also "literature" "review" "anonymisation" "methods" "clinical trials", the first 100 results were screened for each search and relevant results were not found

the techniques used or recommended for data anonymisation in clinical trials, a systematic review is required.

Objective

To identify, describe and synthesise the existing methods/recommendations to anonymise datasets from clinical trials.

Methods

The Preferred Reporting Items for Systematic Review and Meta-Analysis Protocols (PRISMA-P) guidance [10] and the Joanna Briggs Institute Reviewers' Manual: 2015 edition/ supplement/ Methodology for JBI Scoping Reviews [11] were followed for the development of the protocol, where relevant to a methodology systematic review such as this.

Types of publications

We will include publications giving recommendations on anonymising datasets from clinical trials in any therapeutic area. Non-empirical publications such as editorials, expert views or practice guidelines will also be included in this review

Type of Outcomes

The primary outcome is the reported methods and/or recommendations for anonymisation of clinical trials datasets.

Search methods for identification of publications

We will perform a comprehensive systematic search to identify publications reporting methods or recommendations for anonymising clinical trials datasets. No language restrictions will be used. Non-English publications will be initially translated into English using Google

Translate [12]. If they seem relevant, they will be fully translated as local expertise and resources allow, and any literature that we are unable to translate will be declared. The time period of the execution of the searches will be reported

Electronic Searches

Web of Science, Medline (including non-indexed and in-process records), and Embase databases will be searched from inception to the present day.

The search strategy will use the following key concept areas, adopting subject headings and keywords as relevant for each database:

(Clinical) and (trial* or randomi* or research* or control*) and<br>(principle* or guid* or recomm*) and<br>(shar* or reus* or re-us* or access* or open) and<br>(de-identi* or deidenti* or anonym* or privacy or confidential*)

An example of a detailed electronic search strategy is presented in Appendix 1

Searching other resources

In order to ensure the comprehensiveness of our search, the websites of major

- Research governance organisations,
- Public research funding bodies and charities, and
- Academic clinical trials units

will be searched to find guidelines published as grey literature, so as not to omit documents not published as journal articles and not indexed in the bibliographic databases.

To further supplement our search yield, we will use backwards and forward citation searching on the retrieved documents in order to find additional sources. Also, experts and authors known to have published relevant work will be contacted to identify further literature.

Data collection and analysis

Selection of publications

Records will be retrieved and transferred into a reference manager (e.g. EndNote [13] or Citavi [14]), which will be used for de-duplication and to maintain a master library of the records throughout the review process. Two reviewers (AR, CT) will independently screen titles and abstracts for eligibility. Full text copies of all potentially relevant records will be obtained. Two reviewers (AR, CT) will independently assess whether each full text record meets the inclusion criteria. Covidence software [15] will be used for screening. Any discrepancies will be discussed between the reviewers and if agreement cannot be reached then it will be arbitrated by a third reviewer (SCL, CJW or SE)

Publications will be excluded if:

1. They do not have concrete recommendations/methods of anonymisation.
2. Are not from a clinical trial framework
3. Are focused on omics data or big data

Data extraction and management

A data extraction form to collect relevant data items from eligible sources will be developed and piloted in line with Cochrane guidance [16], this will include (but it is not limited to):

- Publication details (Authors names, Journal, year)
- Publication context (Country, main therapeutic area (if applicable))
- All listed recommendations/methods on anonymisation.

Data extraction will be undertaken by one reviewer (AR) who will manually extract relevant data from each included publication onto the data extraction form, which will be sense checked independently by a second reviewer (CT). Any discrepancies will discussed between the reviewers and if agreement cannot be reach then it will be resolved by a third reviewer

(SCL, CJW or SE). Data extracted will be managed and coded using a qualitative research software (e.g. NVivo® [17] or Citavi [14]).

Data synthesis

Data from the included publication will be summarised in descriptive tables.

Results will be summarised by narrative review and if applicable descriptive statistics.

Conclusions

Currently there is a strong demand for academic researchers to share their research data more readily. In clinical trials, data can be shared more widely if they are anonymised, yet we do not have standardised recommendations on how to do this. To the best of our knowledge, this will be the first systematic review of these emerging recommendations/techniques. We will gather and describe all the identified recommendations and we will provide a map for future research regarding anonymisation of datasets in clinical trials to enable data sharing.

Ethics and dissemination

This project will not collect any patient data or outcomes; therefore, it will not be necessary to seek formal National Health Service Research Ethics Committee's approval. However, we will apply for ethical approval from the Internal Ethics Review Board at The University of Edinburgh's Usher Institute. Findings from the review will be presented at scientific conferences and if possible, will be published in a peer-reviewed journal.

Conflicts of Interests

The authors declare no competing interests.

Funding

AR has a scholarship from the University of Edinburgh to undertake a PhD with the support from the Asthma UK Centre for Applied Research (AUKCAR). Neither funder (University of Edinburgh) nor sponsor (AUKCAR) contributed to protocol development. CJW is supported in this work by NHS Lothian via the Edinburgh Clinical Trials Unit.

SCL and CT are supported in this work by their employment at the Edinburgh Clinical Trials Unit.

SE is supported in this work by her employment at the Pragmatic Clinical Trials Unit.

MFD is supported in this work by her employment at the University of Edinburgh.

Author contributions

AR, SCL and CJW conceived the idea for this work supported by SE, MFD and TC. AR wrote the first draft, and all authors contributed to the article.

Appendix 1. Search Strategy

Medline (Ovid)

1. (clinical adj2 (trial* or randomi* or research* or control*)).mp.
2. (principle* or guid* or recomm*).mp. [mp=title, abstract, original title, name of substance word, subject heading word, floating sub-heading word, keyword heading word, protocol supplementary concept word, rare disease supplementary concept word, unique identifier, synonyms]
3. (shar* or reus* or re-us* or access* or open).mp. [mp=title, abstract, original title, name of substance word, subject heading word, floating sub-heading word, keyword heading word, protocol supplementary concept word, rare disease supplementary concept word, unique identifier, synonyms]
4. Data Anonymization/
5. (de-identi* or deidenti* or anonym* or privacy or confidential*).mp. [mp=title, abstract, original title, name of substance word, subject heading word, floating sub-heading word, keyword heading word, protocol supplementary concept word, rare disease supplementary concept word, unique identifier, synonyms]
6. 4 or 5
7. 1 and 2 and 3 and 6

Reference List

1. Song, F., L. Hooper, and Y. Loke, *Publication bias: what is it? How do we measure it? How do we avoid it?* Open Access Journal of Clinical Trials, 2013. **2013**(5): p. 71-81.
2. Berlin, J.A., et al., *Bumps and bridges on the road to responsible sharing of clinical trial data.* Clinical Trials, 2014. **11**(1): p. 7-12.
3. Chan, A.-W., et al., *Increasing value and reducing waste: addressing inaccessible research.* The Lancet, 2014. **383**(9913): p. 257-266.
4. Ohmann, C., et al., *Sharing and reuse of individual participant data from clinical trials: principles and recommendations.* BMJ Open, 2017. **7**(12).
5. El Emam, K. and L. Arbuckle, *Anonymizing health data : case studies and methods to get you started*. 2013: O'Reilly Media, Inc.
6. Hrynaszkiewicz, I., et al., *Preparing raw clinical data for publication trials.* Trials, 2010 **11:9**.
7. Information Commissioner's Office (ICO), *Anonymisation code of practice*. 2012: UK.
8. Keerie, C., et al., *Data sharing in clinical trials - practical guidance on anonymising trial datasets.* Trials, 2018. **19**(1): p. 25.
9. Tudur Smith, C., et al., *Good Practice Principles for Sharing Individual Participant Data from Publicly Funded Clinical Trials*. 2015: UK.
10. Moher, D.S., L.; Clarke, M.; Ghersi, D.; Liberati, A.; Petticrew, M.; Shekelle, P.; Stewart, L.;, *Preferred Reporting Items for Systematic Review and Meta-Analysis Protocols (PRISMA-P) 2015 statement.* Syst Rev, 2015. **4**(1).
11. The Joanna Briggs Institute, *Joanna Briggs Institute Reviewers' Manual: 2015 edition / Supplement*, in *Methodology for JBI Scoping Reviews*. 2015, The Joanna Briggs Institute.
12. Google. *Google Translate*. Available from: https://translate.google.com/.
13. Clarivate Analytics. *EndNote*. Available from: https://endnote.com/.
14. Swiss Academic Software. *CITAVI*. Available from: https://www.citavi.com/en.
15. Covidence. *Covidence*. Available from: https://www.covidence.org/home.
16. Higgins, J.P.T. and S. Green, *Cochrane Handbook for Systematic Reviews of Interventions*. 2011, The Cochrane Collaboration.
17. QSR International. *NVIVO*. Available from: https://www.qsrinternational.com/nvivo/home.
